# Impulse control and related behavioral disorders (ICRD) in Idiopathic Parkinson’s Disease treated with different dopamine agonists in Hong Kong: Is any dopamine agonist better?

**DOI:** 10.1016/j.prdoa.2024.100235

**Published:** 2024-01-05

**Authors:** Hiu Fung Wu, Ellen Lok Man Yu, Bun Sheng, Kwok Kui Wong, Man Au Yeung, Wa Tai Wong, Hon Hang Kwan, Lun Pei Ng, Chun Hin Cheung, Yan Lok Lam, Yat Kwan Chan

**Affiliations:** a Department of Neurology, Medicine and Geriatrics, Princess Margaret Hospital, Hong Kong, China; b Clinical Research Centre, Kowloon West Cluster, Hong Kong, China; c Department of Neurology, Medicine and Geriatrics, Yan Chai Hospital, Hong Kong, China; d Department of Neurology, Medicine and Geriatrics, Pamela Youde Nethersole Eastern Hospital, Hong Kong, China

**Keywords:** Parkinson’s disease, Impulse control disorders, Dopamine agonists, Incidence, Risk factors

## Abstract

**Objective:**

To assess the incidence of Impulse control and related behavioral disorders (ICRD) in Chinese Idiopathic Parkinson Disease (IPD) patients treated with different dopamine agonists (DA), and their clinical characteristics and associated risk factors.

**Methods:**

This was an observational cohort study based on clinical interviews and medical records of IPD patients treated with DA for >6 months in three hospitals in Hong Kong. The short version of Questionnaire for Impulsive-Compulsive Disorders in Parkinson’s Disease (QUIP-S) was used to screen for ICRD. ICRD incidence among different DA, clinical characteristics and risk factors were examined.

**Results:**

Incidence of ICRD was analyzed in 311 patients taking their first, single DA. 43 patients (13.8 %) developed ICRD. The mean duration of IPD was 8.5 ± 5.6 years and median HY stage was 2.5. Bromocriptine and rotigotine users had lower ICRD incidence rate. Both pramipexole [adjusted HR 7.28 (2.46–21.54), p < 0.001] and ropinirole [adjusted HR 6.53 (2.67–15.99), p < 0.001] were independently associated with higher risk of ICRD compared to bromocriptine in multivariate analysis. Similarly, pramipexole and ropinirole appeared to carry higher risk compared to rotigotine but did not reach statistical significance. Male [adjusted HR 2.24 (1.07–4.72), p = 0.033], younger IPD onset [adjusted HR 2.99 (1.44–6.19) for onset < 50 year, p = 0.003] and history of psychiatric disorders [adjusted HR 2.80 (1.39–5.62), p = 0.004] were other independent risk factors.

**Conclusion:**

Bromocriptine and probably rotigotine carried a lower ICRD risk compared to pramipexole and ropinirole.

## Highlights

• This observational cohort study investigated incidence of ICRD among patients using different dopamine agonists in Hong Kong.
• Mean duration of IPD and follow up duration was 8.5 ± 5.6 years.
• Bromocriptine carried a lower ICRD risk compared to pramipexole and ropinirole.
• Rotigotine probably carried a low ICRD risk similar to bromocriptine.

## 1 Introduction

Impulse control and related behavioral disorders (ICRD) including pathological gambling (PG), hypersexuality (HS), compulsive shopping (CS), compulsive eating (CE), punding, hobbyism, walking-about, compulsive medication use (dopamine dysregulation syndrome) are destructive complications in Idiopathic Parkinson Disease (IPD) patients. They are primarily associated with use of dopamine agonists (DA) among other risk factors. Currently, the mainstay of ICRD management in DA users is reduction or cessation of DA with risks of dopamine withdrawal syndrome & worsened motor control.

To date, there is no compelling evidence that risk of ICRD is different across various DA and formulations. There has been no interventional study although lower ICRD rates were reported with long-acting, transdermal [1], [2], [3], [4] or injectable DA [5]. Identifying risk factors and a DA with lower ICRD risk imply new treatment options.

## 2 Methods

### 2.1 Study design

This was an observational cohort study based on clinical interviews and medical records of IPD patients treated with DA in two tertiary hospitals, Pamela Youde Nethersole Eastern Hospital (PYNEH), Princess Margaret Hospital (PMH) and one regional hospital, Yan Chai Hospital (YCH), in Hong Kong. The study received the local ethics committee’s approval.

In PYNEH, IPD patents attending the Parkinson’s disease clinic were included. All attendees were screened routinely and longitudinally with the short version of Questionnaire for Impulsive-Compulsive Disorders in Parkinson’s Disease (QUIP-S) since 2009 [6]. They formed our first cohort. ICRD diagnoses were confirmed by a movement disorder specialist using specific diagnostic criteria [7] as detailed below.

**Fig. 1:**
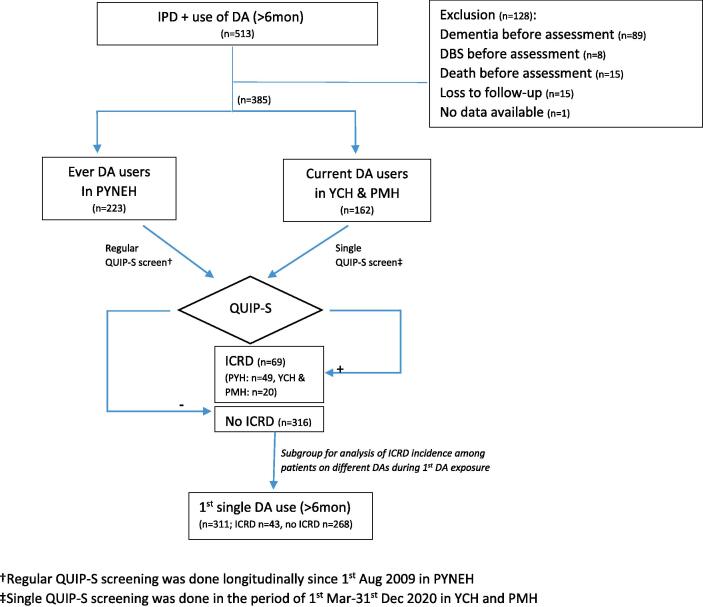
Patients’ recruitment in three hospitals: Pamela Youde Nethersole Eastern Hospital (PYNEH), Princess Margaret Hospital (PMH) and Yan Chai Hospital (YCH), in Hong Kong. IPD, Idiopathic Parkinson Disease. DA, dopamine agonist. ICRD, Impulse control and related behavioral disorders. DBS, deep brain stimulation.

In PMH and YCH, IPD patents attending the neurology clinic were included. Attendees were enquired of any ICRD symptoms during each follow-up by neurologists and neurology trainees. Current DA users undergone a single QUIP-S screening for ICRD from March to December 2020, forming our second cohort. ICRD was confirmed by diagnostic criteria (Fig. 1).

### 2.2 Patients

Inclusion criteria were IPD based on the Queen Square brain bank criteria [8] and ever treatment with DA for more than 6 months.

Exclusion criteria were inability to participate in ICRD assessment due to dementia, death, loss to follow up, and deep brain stimulation (DBS) performed before assessment.

### 2.3 Assessments and measurements

Demographic and clinical features including sex, age of IPD onset, duration of IPD before DA use, history of smoking and psychiatric disorders were collected. Hoehn and Yahr (HY) stage in the “on” state, presence of dyskinesia ever, number and type of DA, dosage and duration of DA, dosage of other anti-parkinsonism drugs, and concurrent psychiatric drugs used were recorded at ICRD onset (for patients who developed ICRD) or at last ICRD evaluation (for patients who didn’t develop ICRD). Levodopa equivalent daily dose (LEDD) [9] was used to express the dosage of DA (DA-LEDD) and other non-DA anti-parkinsonism medications (non-DA LEDD = levodopa LEDD + LEDD of other anti-parkinsonism medications). LEDD conversion factors were as follows: immediate release levodopa: 1; controlled release levodopa: 0.75; entacapone: 0.33; pramipexole: 100; ropinirole: 20; rotigotine: 30; bromocriptine: 10; apomorphine: 10; selegiline oral: 10; selegiline sublingual: 80; rasagiline: 100; amantadine: 1.

ICRD were screened by QUIP-S [10] translated into Chinese. For a positive QUIP-S screening, or when clinical suspicion was high, diagnosis of ICRD was sought according to diagnostic criteria for PG (renamed “gambling disorder” in DSM-V) [7], [11], HS [7], CS (McElroy criteria) [7], CE [7], punding [7], hobbyism [7], compulsive medication use [7]. In patients with confirmed ICRD, the ICRD onset was defined by the date on which ICRD symptoms first appeared according to medical records.

### 2.4 Data analysis

There were three types of DA users. Patients could have been treated with one DA alone (single DA users). They could also have been treated with more than one DAs at the same time (concomitant DA users), or one at a time (sequential DA users).

We aimed to compare the ICRD incidence among different DA and analyze the associated risk factors. Confounding effects from concomitant and previous DA exposure needed to be avoided. Therefore, this analysis was performed only in a subgroup composing of single DA users and sequential DA users, and we examined only the first DA usage (treatment duration of >6 months required) in sequential DA users. This approach had been adopted in another study [1].

ICRD incidence rate was expressed in number-per-1000 drug-years. Fiver-year ICRD-free proportion, shown on a Kaplan-Meier curve (Fig. 2 as esupp), was computed to enable direct statistical comparison between DA. Demographic and clinical characteristics between different DA groups were compared (Table 1). Univariate and multivariate analyses were done to identify independent risk factors associated with ICRD (Table 2). Differential ICRD risk between DA was adjusted for sex, age of IPD onset, duration of IPD before DA, history of smoking, history of psychiatric disorders, HY stage, dyskinesia, non-DA and DA-LEDD in the multivariate analysis.

**Table 1:** Baseline characteristics between DA groups (during 1st DA exposure; n = 311).

		Pramipexole		Ropinirole		Rotigotine		Bromocriptine		p value^a^
		n = 82		n = 89		n = 40		n = 100		
ICRD (%)		12	(14.6)	18	(20.2)	1	(2.5)	12	(12)	0.053
ICRD incidence rate (/1000 drug-yr; 95 % CI)		36.6	(18.9-63.9)	48.9	(29.0–77.3)	7.9	(0.2–44.2)	18.6	(9.6–32.4)	0.016^b^
Male (%)		38	(46.3)	50	(56.2)	22	(55.0)	55	(55)	0.563
Age of IPD onset (yr)		59.7	± 8.4	58.0	± 8.8	63.3	± 11.4	51.8	± 10.6	<0.001^c^
Duration of IPD before DA (yr)		3.1	± 3.9	3.3	± 3.4	7.2	± 6.2	3.4	± 4.9	<0.001^d^
DA duration (yr)		4.0	± 2.1	4.1	± 2.5	3.2	± 2.6	6.5	± 4.3	<0.001^d^
Smoking (%)		16	(19.5)	17	(19.1)	9	(22.5)	9	(9)	0.105
Hx of PsyDis (%)		17	(20.7)	25	(28.1)	12	(30.0)	35	(35)	0.209
Typs of PsyDis										
	Depression (%)	10	(12.2)	15	(16.9)	8	(20.0)	27	(27)	0.078
	Anxiety (%)	13	(15.9)	12	(13.5)	3	(7.5)	11	(11)	0.565
	Others (%)	1	(1.2)	6	(6.7)	4	(10.0)	4	(4)	0.103^e^
HY, median [IQR]		3	[2–3]	3	[2–3]	3	[2–4]	2	[2–3]	0.003^d^
Dyskinesia (%)		25	(30.5)	30	(33.7)	14	(35.0)	56	(56)	0.001
Non-DA LEDD (mg)		506.1	± 413.4	544.3	± 478.9	771.0	± 475.3	716.9	± 508.3	<0.001^d^
DA LEDD (mg)		192.2	± 150.4	125.3	± 66.8	179.4	± 113.1	151.4	± 80.5	0.227^d^
Use of Psy meds										
	Antipsychotics (%)	0		3	(3.4)	6	(15.0)	8	(8)	0.002^e^
	Antidepressants (%)	8	(9.8)	10	(11.2)	6	(15.0)	13	(13)	0.830
	Anxiolytics (%)	12	(14.6)	14	(15.7)	9	(22.5)	13	(13)	0.566
	Others (%)	6	(7.3)	4	(4.5)	3	(7.5)	1	(1)	0.093^e^

Abbreviations CI, confidence interval DA, dopamine agonist IPD, Idiopathic Parkinson Disease PsyDis, psychiatric disorders LEDD, levodopa equivalent daily dose; IQR, interquartile range.

a Pearson's chi-square test, ^b^Likelihood ratio test on Poisson regression model, ^c^one-way ANOVA, ^d^Kruskal-Wallis test, ^e^Fisher’s exact test.

**Table 2:** Univariate and multivariate analyses of ICRD correlates by Cox regression model with frailty term as random effect (during 1st DA exposure; n = 311).

		Univariate			Multivariate		
Variables		HR_unadj_(95 % CI)		p	HR_adj_(95 % CI)		p
1st DA (Ref: Rotigotine)		–			–		
	Ropinirole	7.01	(0.93–52.89)	0.059	4.50	(0.55–36.58)	0.160
	Pramipexole	6.09	(0.78–47.63)	0.085	5.01	(0.59–42.57)	0.140
	Bromocriptine	1.79	(0.23–14.01)	0.581	0.69	(0.08–6.07)	0.736
1st DA (Ref: Bromocriptine)							
	Ropinirole	3.92	(1.77–8.69)	<0.001	6.53	(2.67–15.99)	<0.001
	Pramipexole	3.41	(1.37–8.47)	0.008	7.28	(2.46–21.54)	<0.001
	Rotigotine	0.56	(0.07–4.39)	0.581	1.45	(0.16–12.81)	0.736
Male		1.45	(0.79–2.68)	0.232	2.24	(1.07–4.72)	0.033
Age of IPD onset (below 50 yr)	1.77	(0.95–3.27)	0.071		2.99	(1.44–6.19)	0.003
Duration of IPD (before DA)	0.95	(0.87–1.03)	0.221		0.98	(0.89–1.09)	0.762
Smoking	1.17	(0.54–2.54)	0.690		0.71	(0.29–1.75)	0.462
Hx of PsyDis	2.03	(1.11–3.73)	0.022		2.80	(1.39–5.62)	0.004
HY	0.71	(0.50–0.99)	0.043		0.82	(0.57–1.18)	0.286
Dyskinesia	0.57	(0.29–1.10)	0.094		0.58	(0.25–1.34)	0.201
Non-DA LEDD (per100mg)	0.94	(0.87–1.01)	0.094		0.96	(0.88–1.05)	0.406
DA LEDD (per 100 mg)	1.09	(0.83–1.44)	0.529		1.08	(0.81–1.45)	0.601

Abbreviations: CI, confidence interval; HR, hazard ratio; DA, dopamine agonist; IPD, Idiopathic Parkinson Disease; PsyDis, psychiatric disorders; LEDD, levodopa equivalent daily dose.

The demographic and clinical characteristics of patients in the whole study cohort (containing all three types of DA users) were also reported (Table 3 as esupp).

### 2.5 Statistics

Fisher's exact test and Pearson's chi-square test were used to compare categorical variables. Mann-Whitney *U* test, one-way ANOVA and Kruskal-Wallis test were used to compare numerical variables. ICRD incidence rates between DA were compared using likelihood ratio test on Poisson regression model. Univariate and multivariate Cox regression with a (normally distributed) frailty term as random effect was employed to investigate the association between ICRD and baseline characteristics including the types of first DA used. As our study contained two cohorts, the addition of frailty term as random effect was to account for the correlation due to different collection methods. Statistical analysis was performed by SPSS version 26.0 (Armonk, NY: IBM Corp.). Frailty model was performed by R (version 3.6.1) with package “coxme”. A p < 0.05 was considered as statistically significant.

## 3 Results

513 patients were screened and 128 patients were excluded. In the 385 patients included, 69 patients (17.9 %) developed ICRD. Among the 311 patients who had taken their first DA for more than 6 months, 43 patients (13.8 %) developed ICRD. Mean duration of IPD was 8.5 ± 5.6 years (divided into “IPD duration before DA” and “DA duration”) and median HY stage was 2.5. Ropinirole group had the highest incidence rate (48.9 per 1000 drug-years, 95 % CI 29.0–77.3) while rotigotine group had the lowest (7.9 per 1000 drug-years, 95 % CI 0.2–44.2) (p = 0.016). Five-year ICRD-free proportions for pramipexole, ropinirole, rotigotine and bromocriptine were 0.83, 0.77, 0.97 and 0.95 respectively (Fig. 2 as esupp), which were significantly different among groups (p = 0.005). Pairwise comparison showed bromocriptine users had higher five-year ICRD-free proportion than ropinirole (p = 0.001) and pramipexole (p = 0.014). This pattern was the same for rotigotine users without reaching statistical significance.

The clinical and demographic characteristics of patients in each DA group were shown in Table 1. The groups were significantly heterogenous. In post hoc pairwise comparison analysis (with Bonferroni correction), bromocriptine group had significantly younger IPD onset (p < 0.001), the longest DA duration (p < 0.002) and more dyskinesia than the other three DA. Rotigotine group had the longest IPD duration before DA use (p < 0.001), higher HY (p < 0.02) compared to bromocriptine and ropinirole, and more anti-psychotics and anxiolytics use compared to other three DA. Both bromocriptine and rotigotine groups had higher non-DA LEDD (p < 0.02) compared to other two DA.

In these 311 patients, independent variables associated with ICRD in multivariate analysis were male (adjusted HR 2.24, 95 % CI 1.07–4.72, p = 0.033), younger IPD onset (adjusted HR 2.99 for onset < 50 year, 95 % CI 1.44–6.19, p = 0.003) and history of psychiatric disorders (adjusted HR 2.80, 95 % CI 1.39–5.62, p = 0.004) (Table 2). Between different DA, pramipexole (adjusted HR 7.28, 95 % CI 2.46–21.54, p < 0.001) and ropinirole (adjusted HR 6.53, 95 % CI 2.67–15.99, p < 0.001) were associated with higher risk of ICRD compared to bromocriptine after adjusting for sex, age of IPD onset, duration of IPD before DA, history of smoking, history of psychiatric disorders, HY stage, dyskinesia, non-DA and DA-LEDD. Similarly, pramipexole (adjusted HR 5.01, 95 % CI 0.59–42.57, p = 0.141) and ropinirole (adjusted HR 4.50, 95 % CI 0.55–36.58, p = 0.161) appeared to carry higher ICRD risks compared to rotigotine but did not reach statistical significance.

The clinical and demographic characteristics of our whole study cohort (385 patients) were available in Table 3 (esupp). Ropinirole was the most common DA used. Pathological gambling (n = 24, 34.8 %) and compulsive shopping (n = 23, 33.3 %) were the most frequent ICRD subtype. 25 (36.2 %) of the ICRD patients develop multiple ICRD subtypes. ICRD in 66 (95.7 %) patients either resolved or improved with managements. 17 (24.6 %) of the patients could be kept on same DA dose. None developed dopamine withdrawal syndrome.

## 4 Discussion

Few observational studies were done comparing ICRD risks between different DA. ICRD risk appeared similar across pramipexole [1], [12], [13], [14], ropinirole [1], [12], [13], [14] and pergolide [12], [13], [14]. A study combining reported ICRD prevalence from other studies observed fewer ICRD with bromocriptine [15]. In later preliminary studies, it was suggested that long-acting (extended-release) pramipexole [2], rotigotine [1], [2], [4], or pump apomorphine [5] might be associated with a decreased risk of ICRD, although they were not prospective-controlled studies. As rotigotine was introduced more recently than other non-ergot DA, the number of rotigotine users was smaller and the exposure time shorter in one study [1]. Another study did not employ validated screening tools e.g. QUIP or specific diagnostic criteria for detecting ICRD, which might affect the actual rates of ICRD [2]. Our study had the strength of applying validated screening and diagnostic tool, allowing comparison between all three commonly used non-ergot DA, and achieving similar DA duration between them. It was also one of the few studies in Chinese patients [6], [16], [17], [18], [19].

Incidence of ICRD in our cohort during first DA exposure was 13.8 % (29.2 per 1000 drug-years, 95 % CI 21.2–39.4). Considering the whole cohort, the prevalence was 17.9 %. This was comparable to DOMINION [12] and COPPADIS [4], which also utilized diagnostic criteria after screening questionnaire to avoid overestimation. A few studies reported point prevalence of ICRD in Chinese IPD patients ranging from 3.53 to 31 % [6], [16], [17], [18], [19]. A local study reported 7 % of IPD patients and 10.5 % of IPD patients exposed to bromocriptine had ICRD [6].

We found that the crude incidence rate of ICRD was the highest in ropinirole users, followed by pramipexole, bromocriptine and rotigotine users, in descending order.

Table 1 reflected the prescription pattern of various DA. These differences between the DA groups were adjusted for in the multivariate analysis when we studied the difference of ICRD risks between DA. In short, early DA therapy and the oral DA were preferred in younger patients. In old patients, DA was reserved for late disease and transdermal DA was preferred as the first. This could be seen in the rotigotine group: the patients were the oldest, in a more advanced disease (longer IPD duration, higher non-DA LEDD, the highest HY), had the longest duration of IPD before DA use (non-DA drugs were used first), probably more severe non-motor symptoms (increased anti-psychotics and anxiolytics use without increased frequency of psychiatric disorders). Rotigotine’s advantage in patients with dysphagia and gastroparesis and its relatively limited availability might explain its preference in advanced disease. We also had more data on bromocriptine as historically it was the first DA available in Hong Kong. We saw the longest DA duration, higher non-DA LEDD and most data on young IPD patients (mean age of IPD onset 51.8 yr) in the bromocriptine group. Young IPD onset and higher dopaminergic exposure might be contributors for more dyskinesia in that group.

Our study suggested bromocriptine had a lower hazard ratio for ICRD comparing to pramipexole and ropinirole. To the best of our knowledge, only one longitudinal, observational study included bromocriptine in comparisons of PG prevalence between DA [14]. However, the sample size was small (n = 146). Only 6 patients with PG were identified and it was underpowered. Another study [15], which observed fewer ICRD in bromocriptine, crudely summated prevalence from other observation studies and took the mean. Nonetheless, there is scientific evidence supporting that lower ICRD risk in bromocriptine is possible attributed to its low D3 selectivity. It is believed that selectivity for D2 and D3 receptors (D3 in particular), which are colocalized to the mesocorticolimbic system, leads to more ICRD [15], [20]. Aripiprazole, an atypical antipsychotic with partial D3 agonism leading to ICRD is an example [21]. Pharmacological studies demonstrated various DA’ selectivity for D3 over D2 receptors in the order of: pramipexole, ropinirole, rotigotine, pergolide, apomorphine, bromocriptine, from the highest to the lowest [15], [22]. This trend in D3 selectivity appeared to correlate with the trend of ICRD frequency observed in patients using these DA [15], [21].

Our study also observed that rotigotine had a hazard ratio for ICRD insignificantly lower than pramipexole and ropinirole, but similar to bromocriptine. The initial lower incidence rate and higher five-year ICRD-free proportion in rotigotine users were biased by its prescription pattern in older patients, which was adjusted for in multivariate analysis. Similar patterns for rotigotine were observed before [1], [2]. In COPPADIS, rotigotine was observed to have the lowest ICRD risk, while ropinirole the highest, but not statistically significant. Nevertheless, ropinirole was associated with significantly higher ICRD severity, while rotigotine was not [4]. It had been speculated that the more continuous dopaminergic stimulation provided by rotigotine resulted in fewer ICRD [1]. Transdermal route of administration was known to bypass erratic gastric emptying and gastrointestinal dysmotility, contributing to a more stable plasma drug level and tonic dopaminergic stimulation, resulting in dopamine receptor desensitization. This was supported by preliminary evidence that subcutaneous apomorphine infusion resulted in improvements of ICRD [5], [23]. As for levodopa, continuous, rather than pulsatile, dopaminergic stimulation was also associated with lower prevalence and severity of ICRD [23]. These pharmacological theories arose from clinical observations are incredibly useful for future research. As for dopamine receptor properties, rotigotine carries additional D1 & D5 agonistic action compared to ropinirole and pramipexole. The role of this difference in ICRD development is still unknown.

Concordant with most studies [24], we showed that ICRD were associated with male sex, younger IPD onset and younger age at time of diagnosis.

Finally, pathological gambling and compulsive shopping were the most common subtypes in our cohort, followed by punding or hobbyism. In Hong Kong, lotteries, horse racing, and football gambling are legal and popular. Hong Kong is also known for its convenience of shopping. Comparing to the local study ten years ago [6], PG remained the commonest ICRD but substantially more patients engaged in excessive mobile phone gaming (hobbyism).

Our study had several limitations. It was an ambispective study. Only the patients from PYNEH constituted a longitudinal prospective cohort. If we consider this prospective cohort alone (n = 198), the same pattern of incidence rate of ICRD (i.e. highest in ropinirole users, followed by pramipexole, bromocriptine and rotigotine users, in descending order) was seen. The ICRD rate was significantly lower in bromocriptine users than in ropinirole and pramipexole users in univariate analysis. However, its sample size was not large enough to enter multivariate analysis, thus we had to recruit other patient cohorts. We performed a Cox regression model with a frailty term as random effect to adjust for this difference in collection methods.

As we concentrated on the differential ICRD risks among DA, IPD patients untreated with DA were not included as controls. No comparison in ICRD incidence could be made between these patients, but it had been previously published that 7 % of local IPD patients had ICRD [6]. Limited by the observational design, different DA groups could not be matched, and clinical characteristics were biased by prescription pattern. Assessors were not blinded to the current medications. Diagnosis was made by one physician and interrater variability might exist. Furthermore, currently no ICRD screening tools translated into Chinese were yet validated. We believe the Chinese QUIP-S can soon be validated as in the Korean populations. Also limited by the sample size, we could not evaluate the differential ICRD risk of extended release and immediate release formulations of ropinirole and pramipexole. Finally, we did not measure cumulative DA dose. It could have better represented the exposure in patients with frequent changes of dopaminergic therapies.

No clinical recommendations regarding DA use can be put forward due to our observational design. Still, we feel that prospective studies and randomized controlled trials with a special focus on head-to-head comparison between DA are highly worthwhile. The differential ICRD risk in different DA should continue to receive high attention and priority for research, particularly the possible protective roles of low D3 selectivity and continuous dopaminergic stimulation. Identification or development of a new DA with such properties can represent a new hope.

## 5 Conclusion

Bromocriptine was associated with lower ICRD risk compared to pramipexole and ropinirole. Rotigotine probably carried a similar ICRD risk as bromocriptine. Research should continue to explore the differential ICRD risk in different DA, particularly the role of D3 receptor selectivity and continuous dopaminergic stimulation.

## Funding

This research did not receive any specific grant from funding agencies in the public, commercial, or not-for-profit sectors.

## Declaration of competing interest

The authors declare that they have no known competing financial interests or personal relationships that could have appeared to influence the work reported in this paper.

## References

[1] Garcia-Ruiz P.J., Martinez Castrillo J.C., Alonso-Canovas A., Herranz Barcenas A., Vela L., Sanchez Alonso P., Mata M., Olmedilla Gonzalez N., Mahillo F.I. (2014 Aug). Impulse control disorder in patients with Parkinson's disease under dopamine agonist therapy: a multicentre study. J. Neurol. Neurosurg. Psychiatry.

[2] Rizos A., Sauerbier A., Antonini A., Weintraub D., Martinez-Martin P., Kessel B., Henriksen T., Falup-Pecurariu C., Silverdale M., Durner G., Røkenes Karlsen K., Grilo M., Odin P., Chaudhuri K.R. (2016 Aug). EUROPAR and the IPMDS Non-Motor-PD-Study Group. A European multicentre survey of impulse control behaviours in Parkinson's disease patients treated with short- and long-acting dopamine agonists. Eur. J. Neurol..

[3] Antonini A., Chaudhuri K.R., Boroojerdi B., Asgharnejad M., Bauer L., Grieger F., Weintraub D. (2016 Oct). Impulse control disorder related behaviours during long-term rotigotine treatment: a post hoc analysis. Eur. J. Neurol..

[4] Jesús S., Labrador-Espinosa M.A., Adarmes A.D., Méndel-Del Barrio C., Martínez-Castrillo J.C., Alonso-Cánovas A., Sánchez Alonso P., Novo-Ponte S., Alonso-Losada M.G., López Ariztegui N., Segundo Rodríguez J.C., Morales M.I., Gastón I., Lacruz Bescos F., Clavero Ibarra P., Kulisevsky J., Pagonabarraga J., Pascual-Sedano B., Martínez-Martín P., Santos-García D., Mir P., COPPADIS Study Group (2020). Non-motor symptom burden in patients with Parkinson's disease with impulse control disorders and compulsive behaviours: results from the COPPADIS cohort. Sci. Rep..

[5] Barbosa P., Lees A.J., Magee C., Djamshidian A., Warner T.T. (2016). A retrospective evaluation of the frequency of impulsive compulsive behaviors in Parkinson's Disease patients treated with continuous waking day apomorphine pumps. Mov. Disord. Clin. Pract..

[6] Auyeung M., Tsoi T.H., Tang W.K., Cheung C.M., Lee C.N., Li R., Yeung E. (2011). Impulse control disorders in Chinese Parkinson's disease patients: the effect of ergot derived dopamine agonist. Parkinsonism Relat. Disord..

[7] Voon V., Fox S.H. (2007). Medication-related impulse control and repetitive behaviors in Parkinson disease. Arch. Neurol..

[8] Lees A.J., Hardy J., Revesz T. (2009). Parkinson's disease. Lancet.

[9] Tomlinson C.L., Stowe R., Patel S., Rick C., Gray R., Clarke C.E. (2010). Systematic review of levodopa dose equivalency reporting in Parkinson's disease. Mov. Disord..

[10] Weintraub D., Hoops S., Shea J.A., Lyons K.E., Pahwa R., Driver-Dunckley E.D., Adler C.H., Potenza M.N., Miyasaki J., Siderowf A.D., Duda J.E., Hurtig H.I., Colcher A., Horn S.S., Stern M.B., Voon V. (2009). Validation of the questionnaire for impulsive-compulsive disorders in Parkinson's disease. Mov. Disord..

[11] Diagnostic and Statistical Manual of Mental Disorders (2013).

[12] Weintraub D., Koester J., Potenza M.N., Siderowf A.D., Stacy M., Voon V., Whetteckey J., Wunderlich G.R., Lang A.E. (2010). Impulse control disorders in Parkinson disease: a cross-sectional study of 3090 patients. Arch. Neurol..

[13] Voon V., Hassan K., Zurowski M., Duff-Canning S., de Souza M., Fox S., Lang A.E., Miyasaki J. (2006). Prospective prevalence of pathologic gambling and medication association in Parkinson disease. Neurology.

[14] Bharmal A., Lu C., Quickfall J., Crockford D., Suchowersky O. (2010). Outcomes of patients with parkinson disease and pathological gambling. Can. J. Neurol. Sci..

[15] Seeman P. (2015). Parkinson's disease treatment may cause impulse-control disorder via dopamine D3 receptors. Synapse.

[16] Fan W., Ding H., Ma J., Chan P. (2009). Impulse control disorders in Parkinson's disease in a Chinese population. Neurosci. Lett..

[17] Chiang H.L., Huang Y.S., Chen S.T., Wu Y.R. (2012). Are there ethnic differences in impulsive/compulsive behaviors in Parkinson's disease?. Eur. J. Neurol..

[18] Wang X.P., Wei M., Xiao Q. (2016). A survey of impulse control disorders in Parkinson’s disease patients in Shanghai area and literature review. Transl. Neurodegener..

[19] Zhang Y., He A.Q., Li L., Chen W., Liu Z.G. (2017). Clinical characteristics of impulse control and related disorders in Chinese Parkinson's disease patients. BMC Neurol..

[20] Weintraub D., Claassen D.O. (2017). Impulse control and related disorders in Parkinson's disease. Int. Rev. Neurobiol..

[21] Moore T.J., Glenmullen J., Mattison D.R. (2014). Reports of pathological gambling, hypersexuality, and compulsive shopping associated with dopamine receptor agonist drugs. JAMA Intern. Med..

[22] Gerlach M., Double K., Arzberger T., Leblhuber F., Tatschner T., Riederer P. (2003). Dopamine receptor agonists in current clinical use: comparative dopamine receptor binding profiles defined in the human striatum. J. Neural Transm. (Vienna).

[23] Todorova A., Samuel M., Brown R.G., Chaudhuri K.R. (2015). Infusion therapies and development of impulse control disorders in advanced Parkinson disease: clinical experience after 3 years' follow-up. Clin. Neuropharmacol..

[24] Eisinger R.S., Ramirez-Zamora A., Carbunaru S., Ptak B., Peng-Chen Z., Okun M.S., Gunduz A. (2019). Medications, deep brain stimulation, and other factors influencing impulse control disorders in Parkinson's disease. Front. Neurol..

