# Supplementary material for: Impulse control and related behavioral disorders (ICRD) in Idiopathic Parkinson’s Disease treated with different dopamine agonists in Hong Kong: Is any dopamine agonist better?
Source: Clin Park Relat Disord. 2024 Jan 5;10:100235. doi: 10.1016/j.prdoa.2024.100235 (PMC10825510; doi:10.1016/j.prdoa.2024.100235)

| Table 3 (esupp). Baseline characteristics of IPD patients included according to presence of ICRDs | | | | | | | |  |  |
| --- | --- | --- | --- | --- | --- | --- | --- | --- | --- |
|  |  | All | | Non ICRD | | Ever ICRD | | p value^a^ | |
|  |  | n=385 | | n=316 | | n=69 | |  |  |
| Male (%) | | 205 | (53.2) | 160 | (50.6) | 45 | (65.2) | 0.028 |  |
| Age of IPD onset (yr) | | 56.9 | ± 10.3 | 58.1 | ± 10.4 | 51.4 | ± 8.0 | <0.001 | ^b^ |
| Total DA duration (yr) | | 5.7 | ± 4.6 | 6.0 | ± 4.7 | 4.2 | ± 3.6 | <0.001 | ^b^ |
| Duration of IPD (yr) | | 9.8 | ± 6.1 | 10.3 | ± 6.1 | 7.8 | ± 5.5 | <0.001 | ^b^ |
| Smoking (%) | | 69 | (17.9) | 54 | (17.1) | 15 | (21.7) | 0.362 |  |
| Hx of PsyDis (%) | | 108 | (28.1) | 79 | (25.0) | 29 | (42.0) | 0.004 |  |
| Typs of PsyDis | |  |  |  |  |  |  |  |  |
|  | Depression (%) | 73 | (19.0) | 51 | (16.1) | 22 | (31.9) | 0.003 |  |
|  | Anxiety (%) | 46 | (11.9) | 34 | (10.8) | 12 | (17.4) | 0.124 |  |
|  | Others (%) | 19 | (4.9) | 15 | (4.7) | 4 | (5.8) | 0.758 | ^c^ |
| HY, median (IQR) | | 3 [2-4] | | 3 [2-4] | | 3 [2-3] | | 0.014 | ^b^ |
| Dyskinesia (%) | | 190 | (49.4) | 153 | (48.4) | 37 | (53.6) | 0.433 |  |
| Number of DA use | | 1.4 | ± 0.6 | 1.4 | ± 0.7 | 1.3 | ± 0.6 | 0.484 | ^b^ |
| Number of patients exposed to: | |  |  |  |  |  |  |  |  |
|  | Pramipexole ER+IR (%) | 133 | (34.5) | 114 | (36.1) | 19 | (27.5) | 0.177 |  |
|  | Ropinirope ER+IR (%) | 169 | (43.9) | 134 | (42.4) | 35 | (50.7) | 0.207 |  |
|  | Rotigotine (%) | 88 | (22.9) | 78 | (24.7) | 10 | (14.5) | 0.068 |  |
|  | Bromocriptine (%) | 109 | (28.3) | 87 | (27.5) | 22 | (31.9) | 0.467 |  |
|  | Apomorphine (%) | 2 | (0.5) | 2 | (0.6) | 0 |  | 1.000 | ^c^ |
| Non-DA LEDD (mg) | | 728.1 | ± 566.7 | 737.8 | ± 577.3 | 683.7 | ± 516.6 | 0.568 | ^b^ |
| DA LEDD (mg) | | 164.5 | ± 113.8 | 160.5 | ± 109.0 | 182.6 | ± 133.2 | 0.206 | ^b^ |
| Use of Psy meds | |  |  |  |  |  |  |  |  |
|  | Antipsychotics (%) | 24 | (6.2) | 18 | (5.7) | 6 | (8.7) | 0.407 | ^c^ |
|  | Antidepressants (%) | 50 | (13.0) | 38 | (12.0) | 12 | (17.4) | 0.230 |  |
|  | Anxiolytics (%) | 65 | (16.9) | 49 | (15.5) | 16 | (23.2) | 0.123 |  |
|  | Others (%) | 17 | (4.4) | 14 | (4.4) | 3 | (4.3) | 1.000 | ^c^ |
| ^a^Pearson's chi-square test, ^b^Mann-Whitney U test, ^c^Fisher’s exact test | | | | | |  |  |  |  |
| Abbreviations: DA, dopamine agonist; IPD, Idiopathic Parkinson Disease; PsyDis, | | | | | | |  |  |  |
| psychiatric disorders; LEDD, levodopa equivalent daily dose; ER: extended release | | | | | | |  |  |  |
| IR, immediate release; IQR: interquartile range | | | |  |  |  |  |  |  |

**Figure 2 (esupp).** Kaplan-Meier ICRD-free proportion by year of DA use. 5 year ICRD-free proportion for pramipexole, ropinirole, rotigotine and bromocriptine are 0.828, 0.770, 0.972 and 0.948 respectively, and are signficantly different between groups (p=0.005). Pairwise comparison shows bromocriptine has higher 5 year ICRD-free proportion than ropinirole (p=0.001) and pramipexole (p=0.014). Pairwise comparison shows rotigotine has higher 5 year ICRD-free proportion than ropinirole (p=0.060) and pramipexole (p=0.16), but not reaching statistical significance. DA, dopamine agonist. ICRD, Impulse control and related behavioral disorders.


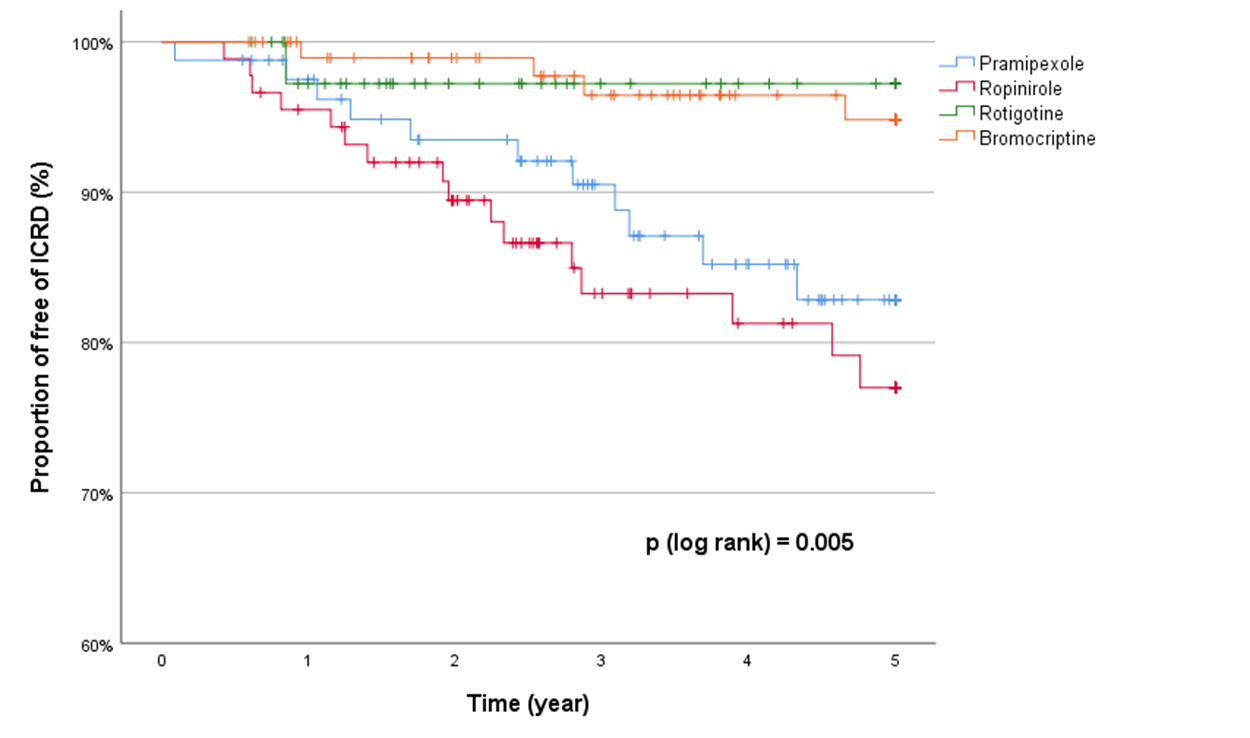

Supplement: Supplementary data 1 [file mmc1.docx]
